# Comparative study on convolutional neural network and regression analysis to evaluate uniaxial compressive strength of Sandy Dolomite

**DOI:** 10.1038/s41598-024-60085-8

**Published:** 2024-04-30

**Authors:** Meiqian Wang, Wenlian Liu, Haiming Liu, Ting Xie, Qinghua Wang, Wei Xu

**Affiliations:** 1 Faculty of Civil Engineering and Mechanics, Kunming University of Science and Technology, Kunming, 650500 Yunnan China; 2 Intelligent Infrastructure Operation and Maintenance Technology Innovation Team of Yunnan Provincial Department of Education, Kunming University of Science and Technology, Kunming, 650500 Yunnan China; 3 Kunming Prospecting Design Institute of China Nonferrous Metals Industry Co., Ltd., Kunming, 650051 Yunnan China; 4 Yunnan Key Laboratory of Geotechnical Engineering and Geohazards, Kunming, 650051 Yunnan China; 5 Faculty of Foreign Languages and Cultures, Kunming University of Science and Technology, Kunming, 650500 Yunnan China; 6 Zhongsheng Civil Engineering (Yunnan) Co., Ltd., Kunming, 650000 Yunnan China

**Keywords:** Sandy Dolomite, Regression analysis (RA), Convolutional neural network (CNN), Uniaxial compressive strength (UCS), Civil engineering, Geology, Petrology

## Abstract

Sandy Dolomite is a kind of widely distributed rock. The uniaxial compressive strength (UCS) of Sandy Dolomite is an important metric in the application in civil engineering, geotechnical engineering, and underground engineering. Direct measurement of UCS is costly, time-consuming, and even infeasible in some cases. To address this problem, we establish an indirect measuring method based on the convolutional neural network (CNN) and regression analysis (RA). The new method is straightforward and effective for UCS prediction, and has significant practical implications. To evaluate the performance of the new method, 158 dolomite samples of different sandification grades are collected for testing their UCS along and near the Yuxi section of the Central Yunnan Water Diversion (CYWD) Project in Yunnan Province, Southwest of China. Two regression equations with high correlation coefficients are established according to the RA results, to predict the UCS of Sandy Dolomites. Moreover, the minimum thickness of Sandy Dolomite was determined by the Schmidt hammer rebound test. Results show that CNN outperforms RA in terms of prediction the precision of Sandy Dolomite UCS. In addition, CNN can effectively deal with uncertainty in test results, making it one of the most effective tools for predicting the UCS of Sandy Dolomite.

## Introduction

The Central Yunnan Water Diversion (CYWD) Project is one of the strategic supporting projects for Yunnan Province to build a radiating center facing South and Southeast Asia, the most significant project out of the 172 major water conservancy and supply projects approved by the Ministry of Water Resources of China, and a key water source project of Yunnan's revitalization strategy. The problem of dolomite sandification in the Yuxi section is one of the major challenges faced by the CYWD Project. Dolomite sandification is a unique geological phenomenon in which dolomite, under the influence of a complex geological environment of multistage tectonic movement, is weathered into silty fine sand, gravel, and/or fragment under the combined action of karstification and weathering, which results in a notable reduction in the rock mass strength and quality^1^. The engineering geological problems caused by Sandy Dolomite are primarily found in the underground engineering projects of transportation and water conservancy industries in Southwest of China^1–4^, as well as the projects in the United States^5^, Canada^6^, Iran^7^, Germany^8^, Egypt^9^ and Italy^10^, etc.

Rock mass strength is one of the most important mechanical properties of rock^11^, which is affected by particle arrangement, discontinuity, saturation, temperature, humidity, and/or weathering^12^. Determining the rock mass strength and deformation is a key field of rock mechanics^13^. To a certain extent, rock mass stability has a substantial effect on human life and property security, and infrastructure construction^14^. It is crucial to evaluate the rock mass strength accurately, efficiently, and reliably, especially in the construction of tunnels and long-distance water diversion projects^15^. As a result, the study of the mechanical characteristics of rock is essential in the field of engineering^16^. The most common method for measuring rock mass strength is the indoor test on the uniaxial compressive strength UCS^17^. The UCS is one of the key parameters of rock mechanics^18^ and one of the most important geomechanical parameters for preliminary and final designs of civil engineering, geotechnical engineering, mining engineering, and underground engineering, i.e., dams, rock excavation, tunnels, slope stability, and infrastructure^19,20^, and is used for the evaluation of rock hardness^21^ and grade classification. Since inaccurate UCS results would cause project budget overruns or even the collapse of related structures^22^, precise UCS calculation of the rock mass is of great significance.

Generally, UCS can be measured by direct or indirect test method. The direct test method refers to the testing of standard samples in the laboratory. The indirect test method serves as the prediction for the UCS based on empirical equation^23^. According to the recommendations of the International Society for Rock Mechanics (ISRM) and the American Society for Testing Materials (ASTM), direct test of UCS necessitates high-quality rock core samples and highly skilled operators, which is costly and time-consuming, making the direct test of UCS impossible in some cases^24,25^. As a result, indirect tests are commonly utilized to predict UCS, such as the Schmidt hammer rebound test^26^, point load test^26,27^, wave velocity test^28^, and needle penetration test^29^, which are easier to perform due to less or no sample preparation, user-friendly equipment, and strong operability in the field. Therefore, the indirect test is a simpler, faster, and more cost-effective measuring method for UCS compared to direct test^30^.

Regression analysis (RA) techniques are frequently used for establishing empirical equations. The researchers have developed many empirical equations for predicting the UCS of rocks. Soft computing-based UCS prediction primarily, i.e., Bayesian analysis^20^, ANN^31,32^, fuzzy system^33,34^, regression trees^35,36^, genetic algorithm^23,37^, imperialist competitive algorithm^38^, and particle swarm optimization technique^39^. So, Soft computing techniques predict UCS more accurately than traditional statistical methods. To overcome the restriction of the prediction precision of RA, this study employed CNN to predict the UCS of Sandy Dolomite. The CNN originated in the 1980s^40^, and then has been widely applied in, i.e., civil and mining engineering and detection^41–46^, rock properties (physico-mechanical properties, chemical compositions, permeability, porosity, rock mass strength, macro and micro image recognition)^47–60^. The CNN-based analysis method for the prediction of the UCS of Sandy Dolomite has not been reported yet.

In this study, based on the testing values of UCS, *SH*, *I*_*s(50)*_, *V*_*p*_, *ρ*, and *n*, this study aims to predict the UCS of Sandy Dolomite by RA and CNN, respectively, and compare the corresponding results.

## Study area and material

**Figure 1:**
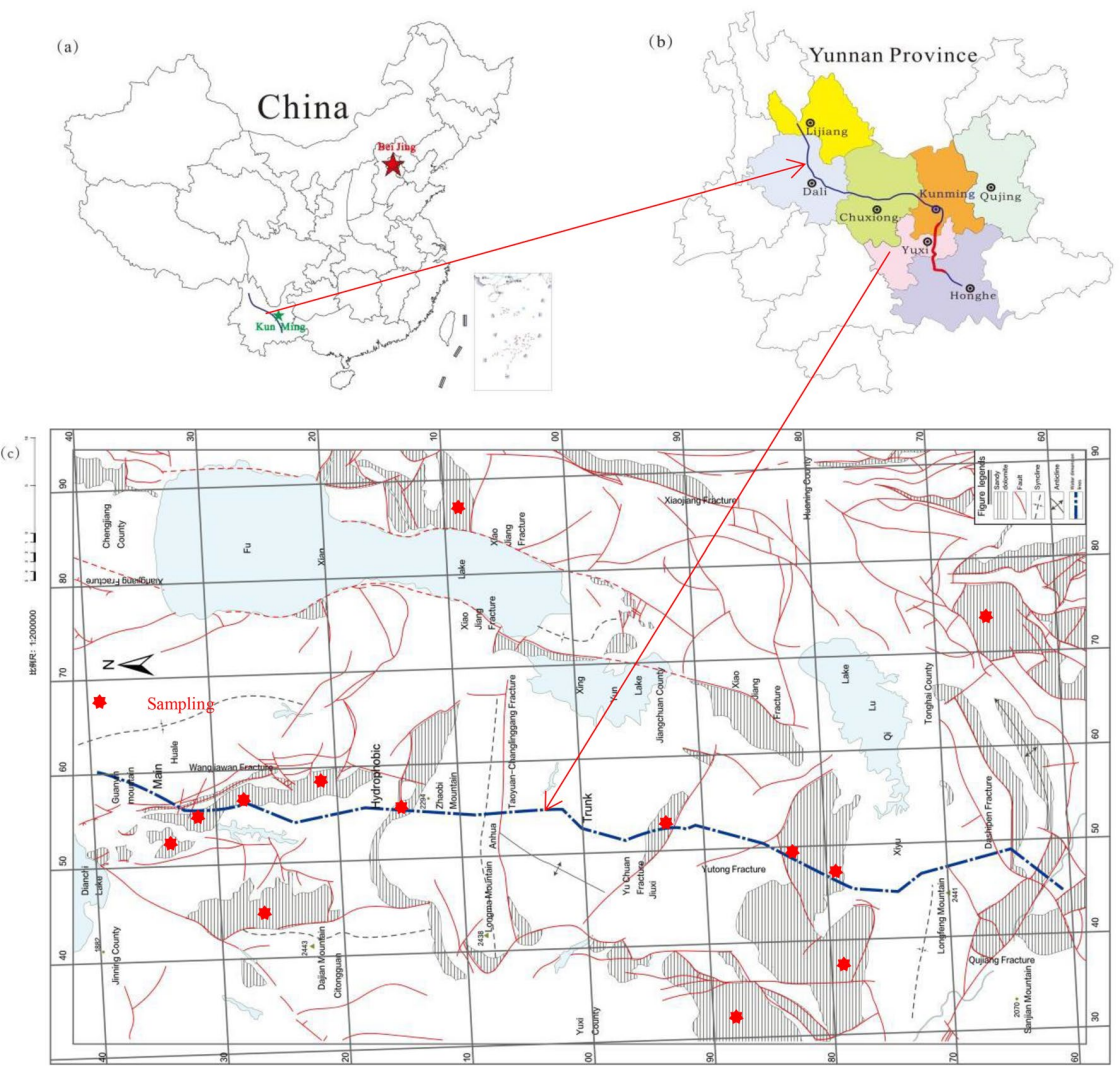
Geological setting of the sampling area and positions of Sandy Dolomites, the construction materials in this study. (**a**) the location of the tunnel of the CYWD Project in China, (**b**) the location of the tunnel of the CYWD Project in Yunnan, (**c**) the location of the tunnel of the CYWD Project in Yuxi section^61^.

The Yuxi Section of the CYWD Project begins from Muyang Village, south of Xinzhuang, Jinning District of Kunming City. The CYWD Project passing through Jinning District and Yuxi City (Hongta District, Jiangchuan District, and Tonghai County). It is part of the plateau mountain area of the central Yunnan basin, with carbonate rocks widely distributed on the surface of the tunnel area. Strong Karst strata are mainly limestone of the Permian Qixia Formation, Maokou Formation (P_1_q + m), and Dalongkou Formation (Pt_1_d) of the Presinian Kunyang Group. Moderate Karst strata are mainly dolomite and limestone of the upper and middle Carboniferous system (C_2+3_), middle Devonian Zaige formation (D3zg), Sinian Dengying formation (Z_b_d_n_), Sinian Doushantuo formation (Z_b_d) dolomite, etc. In this study, the Z_b_dn and Z_b_d Sandy Dolomite along and across the tunnel in the Yuxi section (Fig. 1) are used as the research objects. A total of 158 rock samples were collected from different sites for both field and laboratory tests. RA and CNN are employed in this study to investigate the transformation relation between UCS and *SH*, *I*_*s(50)*_, *V*_*p*_, *ρ*, and *n* based on the PMP of dolomite with different sandification grades, followed by an analysis of the prediction precision and reliability of the two methods.

## Methods

### Porosity and density properties

According to the level of sanding with the differences in color, rock mass structure, microscopic features, alterations, rock mass integrity indexes, rock quality designation (RQD), wave velocity (${V}_{P}$), the intactness index of rock mass (${k}_{v}$), dolomites can be divided into four types of sanding: fierce, strongly, medium and weakly (micro-new rock mass), as listed in Table 1^1,61^.

**Table 1:** Sandy degree of dolomite classification^1,61^.

Sandy degree	Color	Rock organization structure	Volume change	Microscopic feature	Alteration	Rock main characteristics value
Fierce sandy	Uniform discoloration and gloss loss	Totally destroyed and disintegrates and decomposes into loose sand particles	Large	Powder crystal structure	Except for quartz, most of the residual minerals alter to secondary minerals	*K*_v_ < 0.10 *V*_p_ < 1.0 km/s
Strongly sandy	Primary discoloration with part of rock blocks retaining their original color	Mostly destroyed and small part disintegrates and decomposes into loose sand particles	Not small	Fine crystal—medium crystal structure	Except for quartz, feldspar, mica, and femic minerals are already weathered	*RQD* < 20% *K*_v_ = 0.10 ~ 0.15 *V*_p_ = 1.0 ~ 2.0 km/s
Medium sandy	Mostly discoloration and only the fracture of the rock retain slight discoloration as bright color	Mostly appear clear and complete, and the body of rock exhibits fragmentation; small part appears embedded and exhibits fragmental structure	No	Aplitic texture	Femic minerals exhibit oxidation and corrosion; feldspar exhibits opacification	*RQD* = 20% ~ 40% *K*_v_ = 0.15 ~ 0.35 *V*_p_ = 2.0 ~ 3.5 km/s
Weakly sandy	Uniform slight discoloration as bright color	All appear original withcomplete organization structure	No	Medium crystal structure	Only the part along with the crack appear the phenomenon of weathering and alteration or/and the immersion of the corrosion	*RQD* > 50% *K*_v_ > 0.40 *V*_p_ > 4.0 km/s

**Figure 2:**
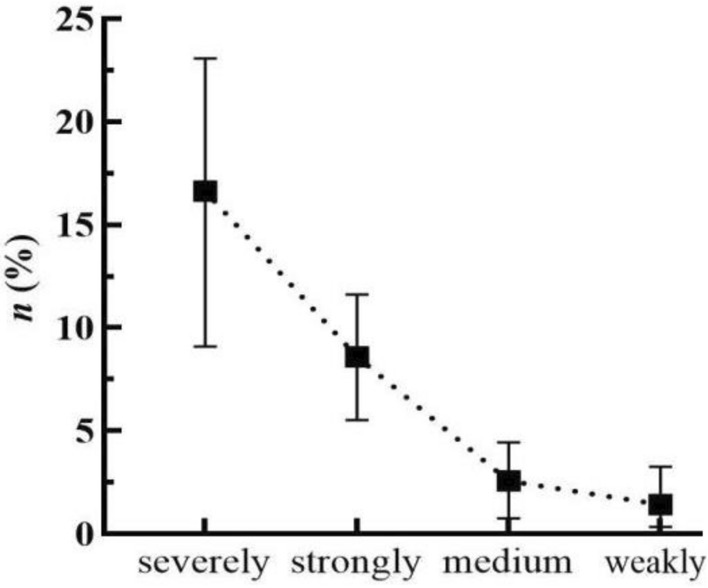
Relationship between Sandy Dolomite and *n.*

**Figure 3:**
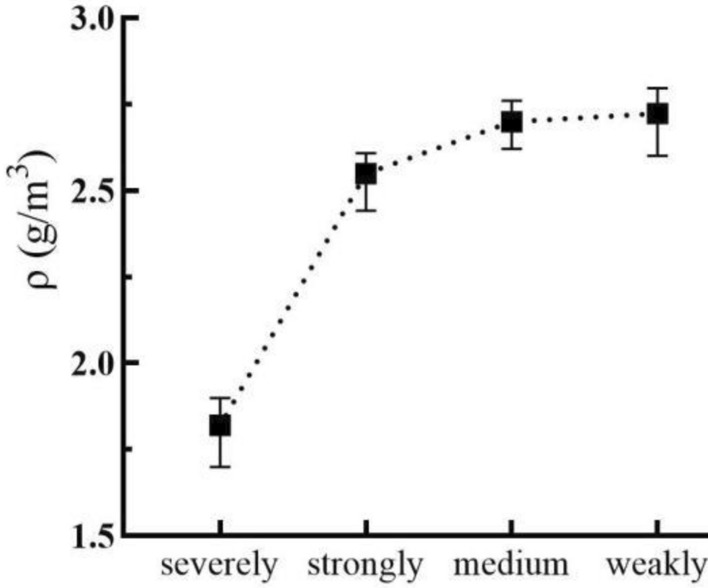
Relationship between Sandy Dolomite and *ρ.*

In this study, the Sandy Dolomite along and near the Yuxi section of the CYWD Project was taken as the research object. Dolomite with various sandification grades was sampled in field outcrops, boreholes, and tunnels to determine the PMP. The test results are displayed in Table 2, Figs. 2, and 3.

**Table 2:** Statistics of *ρ* and *n* of Sandy Dolomite.

Sandy Dolomite	Number of tests	ρ (g/m^3^)			n (%)		
		Min	Max	Mean	Min	Max	Mean
Fierce	19	1.7	1.9	1.82	9.09	23.08	16.63
Strongly	24	2.442	2.608	2.549	5.53	11.62	8.59
Medium	90	2.62	2.761	2.699	0.77	4.45	2.577
Weakly	68	2.601	2.797	2.723	0.34	3.26	1.426

According to Matula’s method^62^, fierce Sandy Dolomite is a rock with low density and high porosity; strongly Sandy Dolomite is a rock with medium density and porosity; medium and weakly Sandy Dolomite is a rock with high density and low porosity.

According to Figs. 2 and 3, the higher the sandification grade increases, the higher porosity and the lower density of Sandy Dolomite, indicating that the sandification grade increases with the rise of porosity and the decline of density.

**Figure 4:**
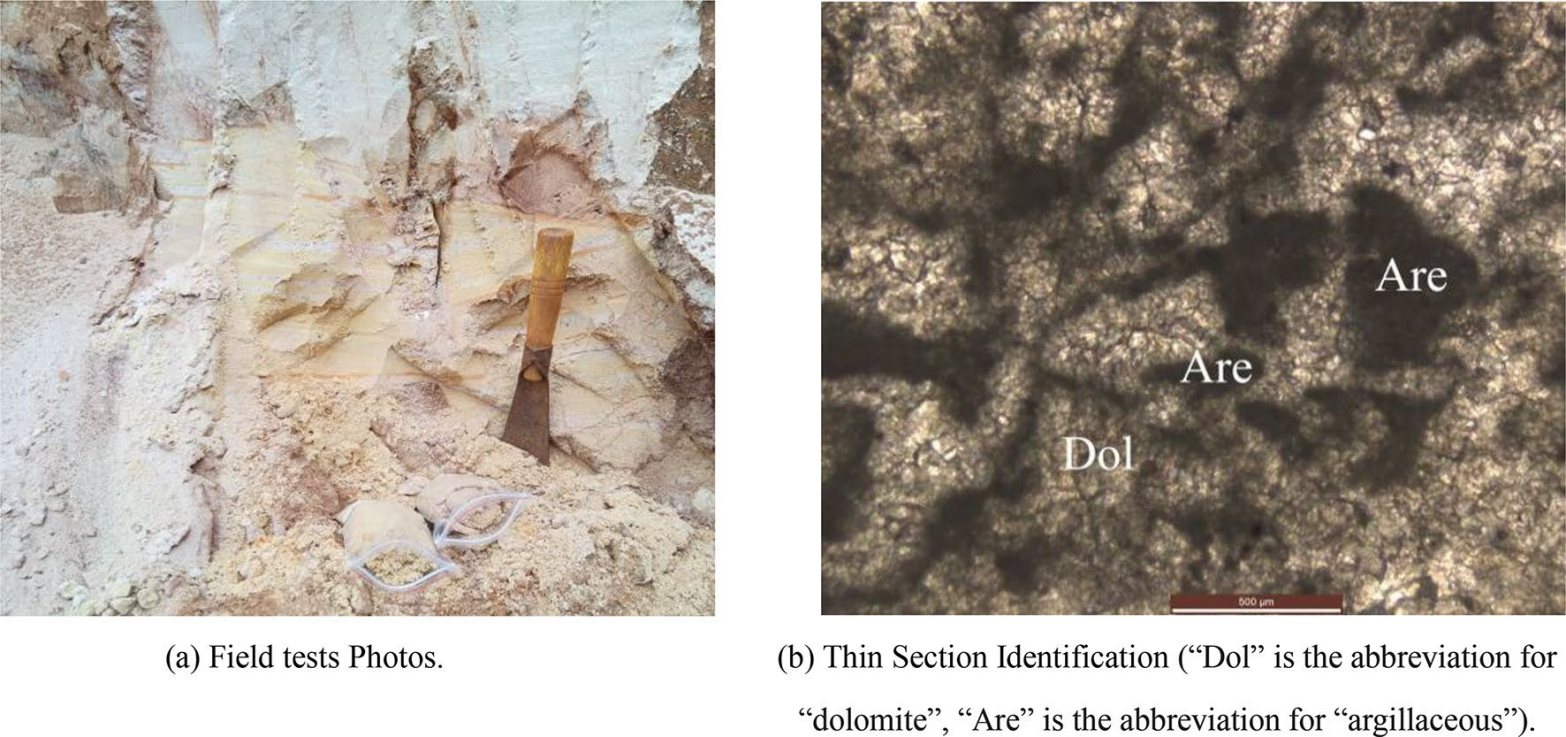
Fierce Sandy Dolomite (E 102°41′38.34″, N 24°35′48.82″). (**a**) Field tests Photos. (**b**) Thin Section Identification (“Dol” is the abbreviation for “dolomite”, “Are” is the abbreviation for “argillaceous”).

Since the fierce Sandy Dolomite can be easily crushed by hand, a complete rock block cannot be obtained for mechanical testing, as shown in Fig. 4a. The thin rock section identification is shown in Fig. 4b. The indoor size distribution test result shows that the fierce Sandy Dolomite is in a state of silty fine sand.

**Figure 5:**
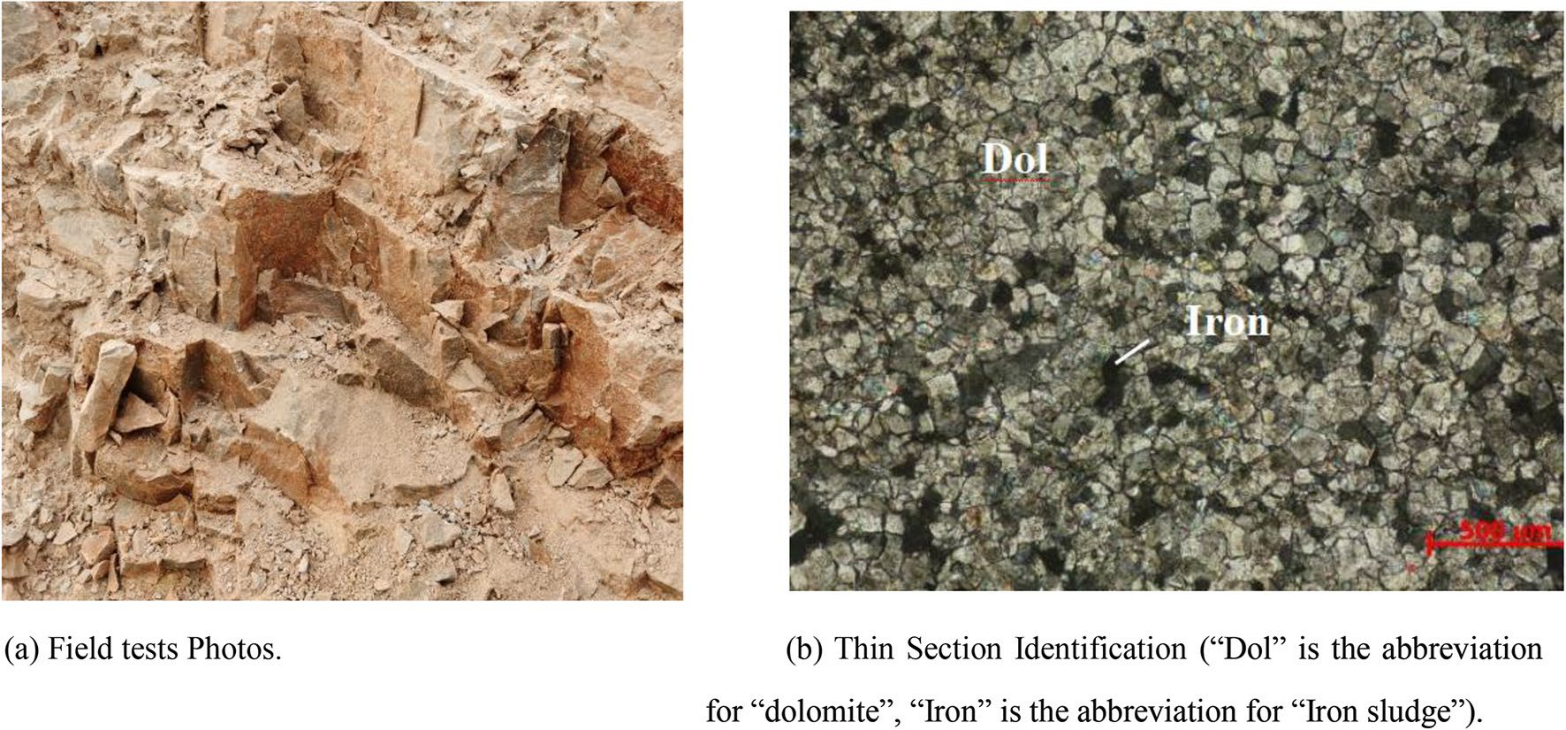
Strongly Sandy Dolomite (E 102°39′48.92″, N 24°15′33.16″). (**a**) Field tests Photos. (**b**) Thin Section Identification (“Dol” is the abbreviation for “dolomite”, “Iron” is the abbreviation for “Iron sludge”).

Since the rock mass strength of strongly Sandy Dolomite is obviously weakened, with forming weathering fissures, a complete rock block cannot be obtained for mechanical testing, as shown in Fig. 5a. The thin rock section identification is shown in Fig. 5b. Schmidt rebound test on the strongly Sandy Dolomite sample shows that there is no rebound reading, indicating that the rock mass has been damaged.

In view of the aforementioned condition, this study only took the medium and weakly Sandy Dolomite as the research objects and analyzed the relationship between UCS and *SH*, *I*_*s(50)*_, *V*_*p*_, *ρ*, and *n*. The PMP of rock samples were tested using the methods specified by ISRM by establishing at least six sets of samples for each rock sample and calculating their average value.

### Uniaxial compressive strength test

**Figure 6:**
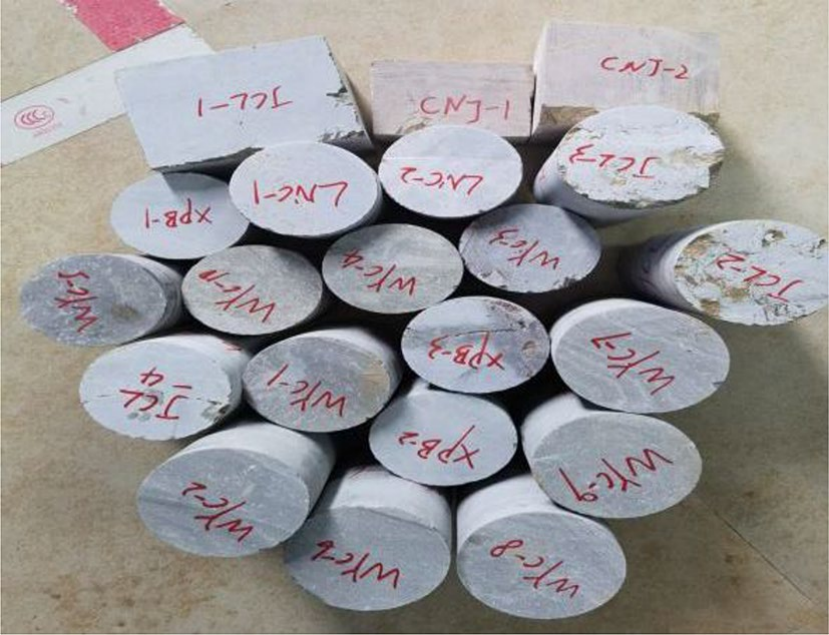
Processed partially Sandy Dolomite core sample.

**Figure 7:**
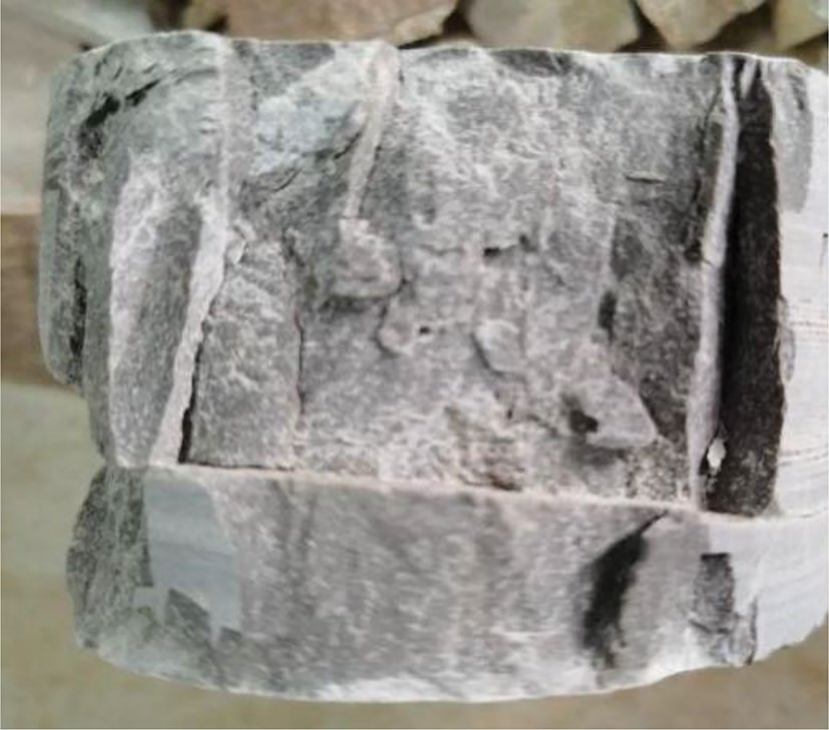
Rock core after UCS test.

Rock core samples were cut using an automatic double-blade rock core cutting machine (SCQ-4A). The diameter of cylindrical core samples ranges from 44.35 to 65.40 mm, and the length ranges from 42.27 to 105.35 mm, with a length/diameter ratio of 0.66 ~ 2.17, as shown in Fig. 6. According to ISRM^63^, the dolomite with different sandification grades were tested, the average value was taken as the UCS of the sample, and the length/diameter ratio of UCS should be 2.0 (50 mm × 100 mm)^64^, which is not a standard size according to the ratio suggested by the ISRM^63^ (> 2.5), Eq. (1) was adopted to convert the tested UCS* to UCS^65^. The sample should be loaded using an electro-hydraulic loading testing machine (HYE-2000), with the loading rate controlled between 1000 N/s and 2000 N/s, and a maximum loading capacity of 2000 kN. The rock core after the loading is shown in Fig. 7.

1 $$ UCS = \frac{{0.8668UCS^{*} }}{{0.778 + \frac{0.222}{{L/D}}}} $$

where L is length, D is diameter, and UCS* is the UCS of the specimen at a ratio of L/D.

### Schmidt hammer rebound test

Schmidt hammer was originally invented to test the strength of concrete^66^ and widely used as an indicator test for characterizing rock mass strength and deformation owing to its rapidity and ease of use, simplicity, portability, low cost and non-destructive nature^67^. Taking into account the impact factors, i.e., grain size of rock mass, anisotropy, sample size, weathering, and water content, its recommended method was improved in ISRM by Aydin^68^.

Although the prediction accuracy of UCS could be improved via the Schmidt rebound test, there still are some shortcomings with RA. RA is based on limited experimental data sets and specific rock types, so some empirical formulas couldn’t be applied generally in engineering practice. Obviously, the type of empirical formula of RA was chosen by academics objectively, and the outcomes are not appropriate enough. There are also some limitations with ANN. The data processing procedure is not clear or sufficiently transparent due to the hidden layer in the ANN is a black box, and the neural network may misunderstand the purpose of the researchers, so the prediction result should be verified frequently^26^.

**Figure 8:**
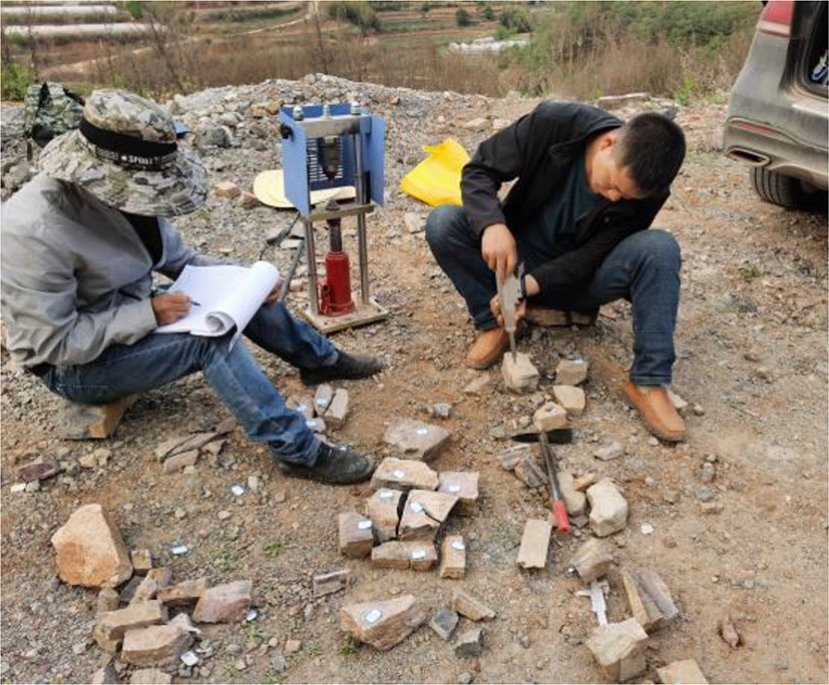
Field tests of SH test.

In this study, the HT-225 Schmidt hammer is utilized to calculate the rebound value, with the rebound test repeated 25 times for each sample. After removing the 5 maximum and 5 minimum values, the average of the remaining values was taken as the final result, and the field tests is shown in Fig. 8. Moreover, the minimum thickness of medium and weakly Sandy Dolomite is 110 mm and 75 mm according to the SH test results, respectively.

### Point load test

The point load tester is portable and suitable for all types of rocks, and the sample of PLT need not to be cut and grind in the field or laboratory^27^. Protodyakonov^69^ first put forward the idea of PLT with irregular blocks, and then D'Andrea^70^ and Franklin^71^ studied the transformation between rock's I_s(50)_ and UCS. The point load tester can be used in various working conditions, i.e., outcrops, exploration pits, adits, roadways, and other caverns. The tested sample can be cylindrical, irregular shaped^72^, or unprocessed, filling the blanks in soft and broken rock mass strength tests.

**Figure 9:**
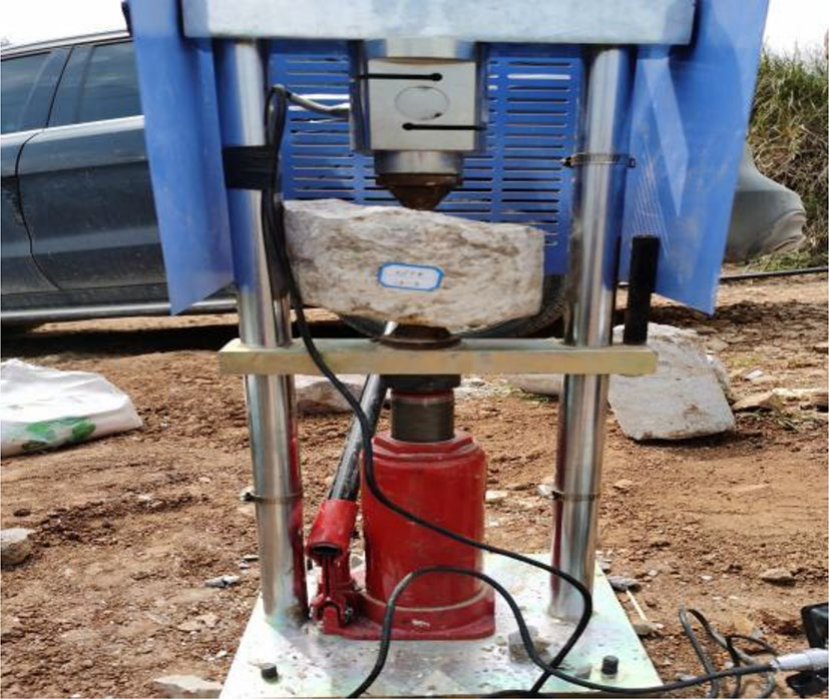
Field tests of PLT.

The PLT was performed only on the irregular block samples in this study (Fig. 9). The irregular sample can be calculated by utilizing the method of equivalent core diameter by ASTM standards, and I_s(50)_ was determined by Eq. (2).

2 $$ \begin{gathered} D_{{\text{e}}}^{2} = \frac{4 \cdot W \cdot D}{\pi } \hfill \\ I_{{{\text{s}}50}} = \frac{4 \cdot P}{{D_{e}^{2} }} \hfill \\ \end{gathered} $$

where P is the failure load and De is the equivalent diameter of irregular blocks; D and W are of the maximum length and average width of the failure surface in millimetres.

### Compressional wave velocity test

The compressional wave velocity, depends on mineral composition, texture, fabric, weathering degree, water content, and rock density^22,33,39,73^, is calculated according to the transmission time between the transmitted and received wave, which is non-destructive and is also feasible in field or laboratory.

### Clarify

The person in Fig. 8 is the author of this article, the recorder is the first author Meiqian Wang, and the experimenter is the fifth author Qinghua Wang.

## Model analysis

### Regression analysis

SPSS26.0 was utilized to investigate the relationship between UCS and *I*_*s(50)*_, *SH*, *V*_*p*_, *ρ*, and *n* based on the RA model. F-test and T-test with 95% confidence intervals were applied to confirm the model's dependability. The results demonstrated that the RA model has a wide variety of applications. The UCS value can be predicted simply by taking the parameters of most frequent rock properties as the input parameters of the RA model.

R^2^ refers to the proportion of the regression sum of squares in the entire sum of squares, which is used for assessing the effectiveness of model. R^2^ ranges from 0 to 1. The closer R^2^ is near to 1, the higher the model's goodness is of fitness. The variance analysis of the entire regression equation is equal to the R^2^-based hypothesis testing on the goodness of fit of the regression equation.

### Convolutional neural network

The CNN consists of neurons with learnable weights and bias vectors. Compared with ANN, CNN adopts a mathematical operation called convolution, the traditional matrix multiplication of ANN was replaced by the mathematical operation in the network structure. The convolution operation effectively utilizes the two-dimensional structure of the input parameters, thus obtaining superior calculation outcomes.

**Figure 10:**
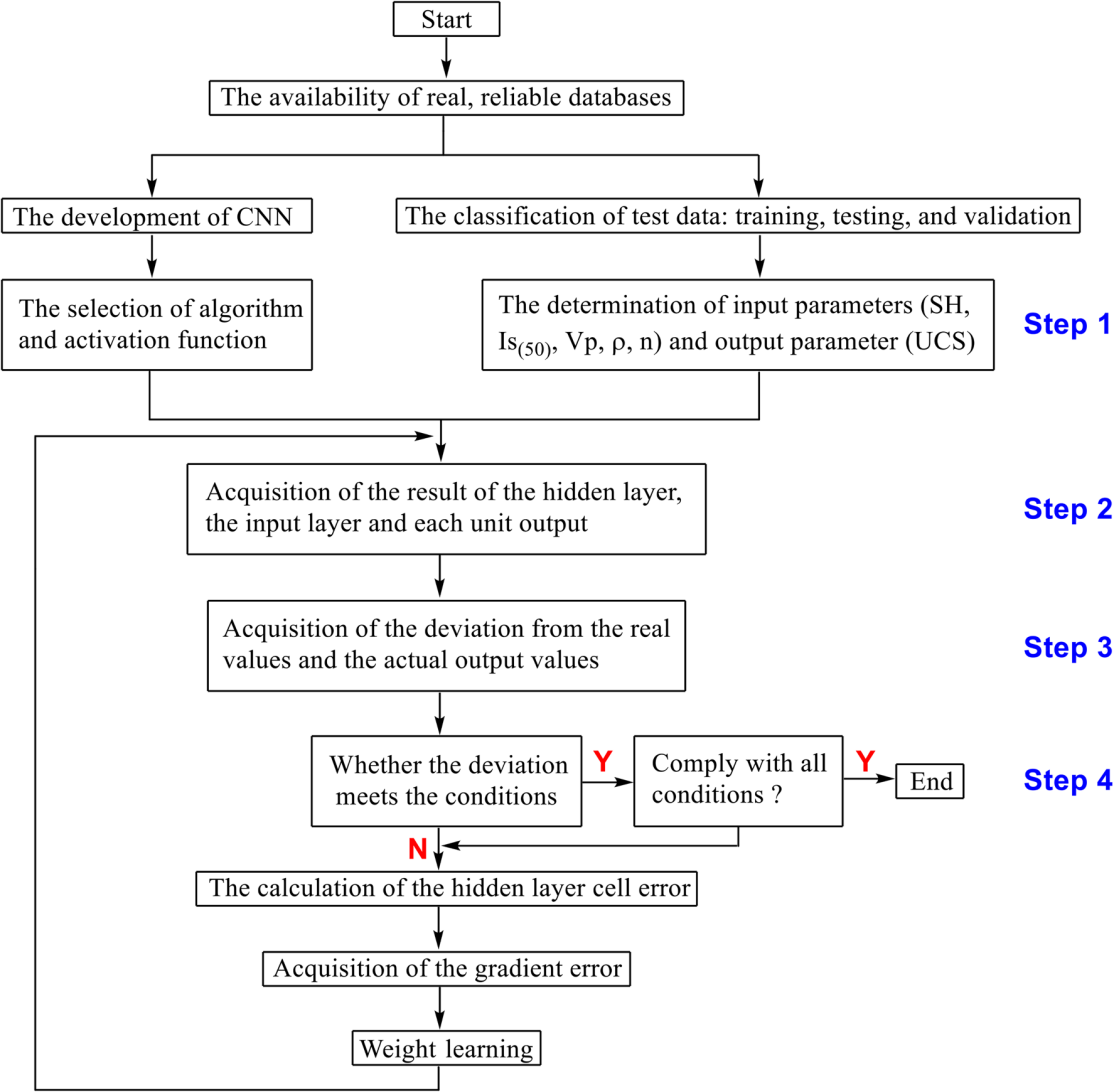
Flow chart for USC prediction based on CNN from index properties of rock.

Similar to conventional neural networks, CNN has two operational states, including the training and testing phases. The parameters are continuously optimized during the training phase in the learning phases of CNN, and the brand new dataset is used to evaluate the learning ability of the completed CNN in the testing phases (Fig. 10).

**Figure 11:**
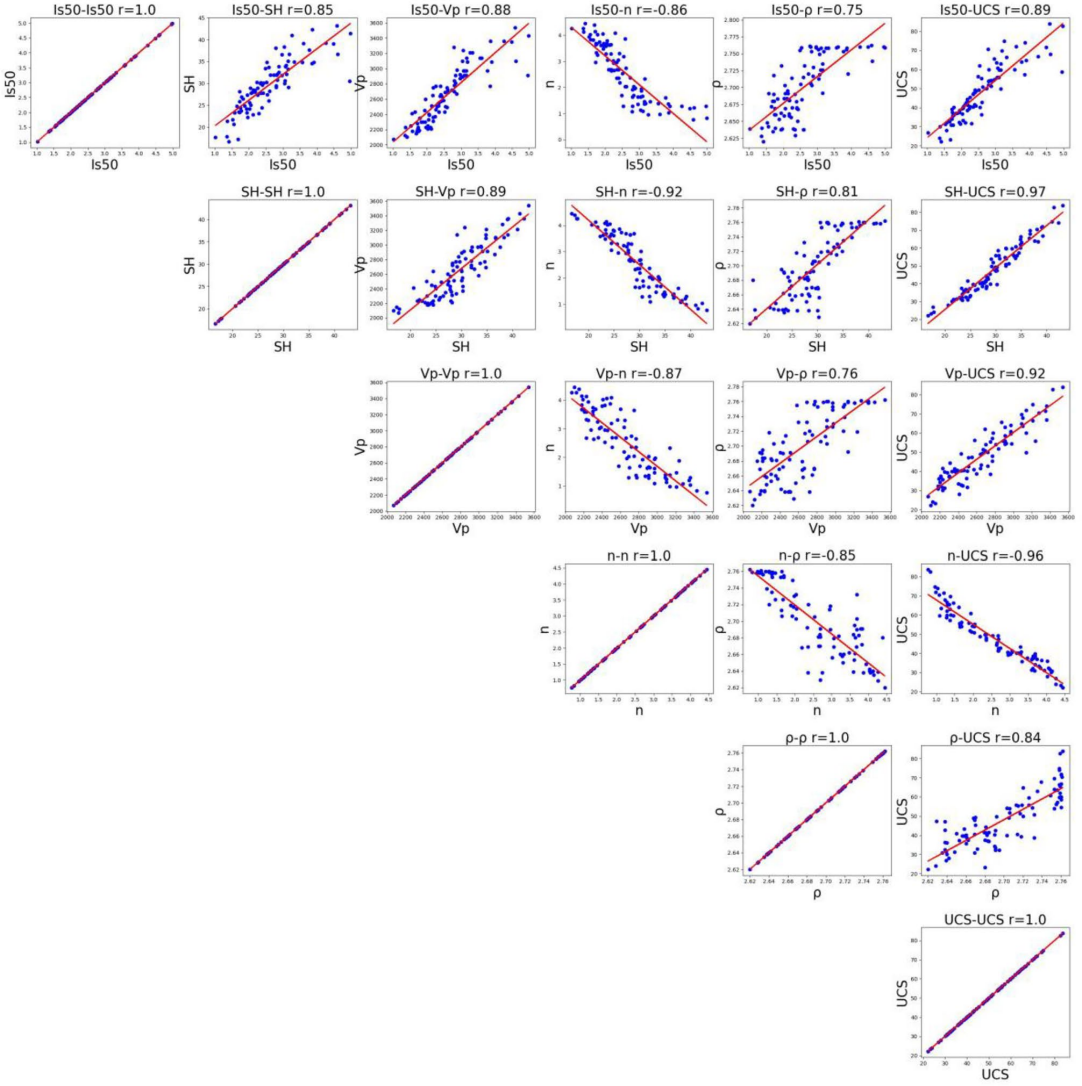
Scatterplot of medium Sandy Dolomite in terms of UCS, *SH*,* I*_*s(50)*_,* V*_*p*_, *ρ*, and *n.*

As shown in Fig. 11, the procedure contains four steps, which are discussed in detail below^54^.

*Step 1* Take a sample randomly from the data set and record it as *X*_*p*_ and *Y*_*P*_, and input *X*_*p*_ into the network as the input parameter. The *Y*_*p*_ serves as the reference value of the result.

*Step 2* Obtain the corresponding output *Q*_*P*_ through hierarchical calculation.

The calculation of CNN works base on the dot product of the input value and the weight matrix of each layer, and the final output value is obtained after operation layer by layer, as shown in Eq. (3).

3 $$ Q_{P} = F_{n} ( \cdot \cdot \cdot F_{2} (F_{1} (X_{P} W^{1} )W^{2} ) \cdot \cdot \cdot )W^{N} $$

*Step 3* Calculate the deviation between the output value *Q*_*P*_ and the corresponding true value *Y*_*P*_.

*Step 4* The weight matrix is adjusted through the back-propagation algorithm by minimizing the error.

In the *K*th layer, the weights of the *i*th neuron(*i* = *1, 2*,…, *n*) can be written as *W*_*i,1*_*, W*_*i,2*_,…, *W*_*i,n*_. First, the weight coefficient *W*_*i,j*_ should be set to a random number close to zero, allowing the gradient descent algorithm to find the local optimal solution. The training samples *X* = (*X*_*1*_*, X*_*2*_,…,* X*_*n*_*)* are inputted, and the corresponding real outputs *Y* = (*Y*_*1*_*, Y*_*2*_,…* Y*_*n*_) are obtained. Similarly, the real outputs of each layer can be calculated through weight calculation, as shown in Eq. (4–5):

4 $$ U_{i}^{k} = \sum\limits_{j = 1}^{n + 1} {W_{i,j} } X_{i}^{k - 1} ,\left( {X_{n + 1}^{k - 1} = 1,W_{i,n + 1} = \theta_{i} } \right) $$

5 $$ X_{{\text{i}}}^{k} = f(U_{i}^{k} ) $$

where $X_{{\text{i}}}^{k}$ refers to the output of the *i*th neuron in the *K*th layer; $W_{i,n + 1}$ represents the threshold value $\theta_{i}$. The desired and real outputs can be used to calculate the learning error $d_{{\text{i}}}^{k}$ of each layer, as well as the corresponding errors of the hidden and output layers. If the output layer is recorded as *m*, the corresponding expression is shown in Eq. (6):

6 $$ d_{{\text{i}}}^{k} = X_{i}^{m} (1 - X_{i}^{m} )(X_{i}^{m} - Y_{i} ) $$

Based on the gradient error calculation, the learning error of other layers can be calculated by Eq. (7):

7 $$ d_{{\text{i}}}^{k} = X_{i}^{k} (1 - X_{i}^{k} )\sum\limits_{l} {W_{lj} } d_{{\text{i}}}^{k + 1} $$

Judge whether the error meets the calculation condition. If YES, the algorithm ends. Otherwise, the algorithm continues by modifying the weight coefficients through learning error, as shown in Eq. (8).

8 $$ W_{ij} (t + 1) = W_{ij} (t) - \eta \cdot d_{i}^{k} \cdot X_{j}^{k + 1} + \alpha \Delta W_{ij} (t) $$

where $\Delta W_{ij} (t) = - \eta \cdot d_{i}^{k} \cdot X_{j}^{k - 1} + \alpha W_{ij} (t + 1) = W_{ij} (t) - W_{ij} (t - 1)$; $\eta$ refers to the learning rate. The modified weight values will be used to calculate the real outputs until the error meets the conditions.

### Advantages and performance evaluation of CNN

Compared with ANN, CNN is superior for the following reasons: (1) the introduction of the receptive field, also known as the local connection. Each neuron only receives connections from a small number of neurons in the upper layer, which only perceives data from local rather than all input dimensions. (2) The introduction of weights. Each neuron functions as a filter, using a fixed convolution kernel for the convolution of the entire set of inputs. (3) The introduction of multiple convolution kernels. By adding the channel dimension, CNN can extract more features. In addition to the global data distribution, multiple convolution kernels are conducive to better modeling the correlation between local features in the condition of complex data analysis, which could be fitter various nonlinear mappings.

The prediction of UCS is a research topic in the field of RA. Five statistical indices are used for evaluating the performance of the prediction model in RA and CNN training, including MAE, MSE, RMSE, MAPE, and R^2^. The corresponding mathematical expressions of all indices are shown in Eq. (9)–(13) ^74,75^:

9 $$ MAE = \frac{1}{m}\sum\limits_{i = 1}^{m} {\left| f \right._{{\text{i}}} } - \left. {f_{UCS,i} } \right| $$

10 $$ MSE = \frac{1}{m}\sum\limits_{i = 1}^{m} {(f_{i} } - f_{UCS,i} )^{2} $$

11 $$ RMSE = \sqrt {\frac{1}{m}\sum\limits_{i = 1}^{m} {(f_{i} } - f_{UCS,i} )^{2} } $$

12 $$ MAPE = \frac{100\% }{{\text{m}}}\sum\limits_{i = 1}^{m} {\left| {\frac{{f_{i} - f_{UCS,i} }}{{f_{UCS,i} }}} \right|} $$

13 $${{\text{R}}}^{2}=1-\frac{{\sum ({f}_{i}-{f}_{UCS,i})}^{2}}{{\sum ({f}_{i}-\overline{f })}^{2}}$$

where *f*_*i*_ and *f*_*UCS,i*_ refer to the measured and predicted values of UCS, respectively. $\overline{f }$ refers to the average of the measured UCS values. *m* is the number of the measured values of UCS.

In the CNN-based prediction method, statistical indices are employed to assess the effectiveness of the model. The input parameters are ranked according to the contribution of each parameter in the prediction of UCS. If one input parameter results in high MSE, MAE, MAPE, RMSE, and low R^2^, it means that the deleted parameter has a significant influence on CNN.

## Results and discussion

Two groups of real UCS data from Sandy Dolomite along and near the Yuxi Section of the CYWD Project were used for research object in this study. A total of 158 rock samples were collected and tests, and 158 groups of UCS and *SH*, *I*_*s(50)*_, *V*_*p*_, *ρ*, and *n* data were obtained.

### Analysis results of medium Sandy Dolomite

The UCS of medium Sandy Dolomite was taken as the dependent variable, and *SH*, *I*_*s(50)*_, *V*_*p*_, *ρ*, and *n* were taken as the independent variables. SPSS 26.0 was used for fitting linear RA to obtain the relationship between the dependent and independent variables [Eq. (14)]. This method is obtained through multiple RA, and the used data in this method were considered the relationship between parameters. The equation with the highest R^2^ was chosen as the empirical equation after various equations have been taken into account.

14 $$ {\text{UCS}} = {1}.{\text{21I}}_{{{\text{s}}({5}0)}} + {1}.0{\text{9SH}} + 0.00{\text{6Vp}} - {4}.{\text{528n}} + {6}.{889}\rho - {9}.{471} \;\;\;\;{\text{R}}^{{2}} = 0.{973} $$

Equation (14) shows a good correlation between the PMP (*SH*, *I*_*s(50)*_, *Vp*, *ρ*, *n* and UCS) of medium Sandy Dolomite, with the R^2^ of 0.973, indicating the goodness of fitness of the RA.

In addition to the descriptive statistics of UCS, *SH*, *I*_*s(50)*_, *V*_*p*_, *ρ*, and *n*, the Pearson correlation coefficients between each pair of variables were calculated based on the database (Fig. 11). The five parameters are highly correlated with UCS, which implied that they have a significant correlation in predicting UCS, and the correlation matrix is shown in Table 3.

**Table 3:** Correlation matrix between UCS and PMP.

	I_s(50)_	SH	Vp	n	ρ	UCS
I_s(50)_	1.000000					
SH	0.846322	1.000000				
Vp	0.882991	0.885593	1.000000			
n	− 0.857435	− 0.918388	− 0.874688	1.000000		
ρ	0.751594	0.812292	0.756019	− 0.851650	1.000000	
UCS	0.886514	0.965271	0.919986	− 0.960027	0.841166	1.000000

The synergetic coefficient between UCS and *SH*, *I*_*s(50)*_, *V*_*p*_*,* and *ρ* of medium Sandy Dolomite is positive, which demonstrate a positive correlation between UCS and these four parameters (*SH*, *I*_*s(50)*_, *Vp*, *ρ*) and that UCS increases with the rise of four parameters. The synergetic coefficient between UCS and n is negative, which demonstrate that there is a negative correlation between the two, and UCS decreases with the rise of *n* (Fig. 11 and Table 3). The UCS of medium Sandy Dolomite, five parameters exhibits good prediction performance, among which the most optimal indices are *SH*, *I*_*s(50)*_, and *V*_*p*_ [Eq. (14) and Table 2].

### Analysis results of weakly Sandy Dolomite

The UCS of medium Sandy Dolomite was taken as the dependent variable, and *SH*, *I*_*s(50)*_, *V*_*p*_, *ρ*, and *n* were taken as the independent variables. SPSS 26.0 was used for fitting linear RA to obtain the relationship between the dependent and independent variables [Eq. (15)].

15 $$ {\text{UCS}} = {6}.{\text{36I}}_{{{\text{s}}({5}0)}} + 0.{\text{631SH}} + 0.0{\text{19Vp}} + 0.{\text{566n}} - {1}.{4}0{5}\rho - {5}0.{784}\;\;\;\;{\text{R}}^{{2}} = 0.{795} $$

Equation (15) shows a good correlation between the PMP (*SH*, *I*_*s(50)*_,*Vp*, *ρ*, *n* and UCS) of weakly Sandy Dolomite, with the R^2^ of 0.795, indicating the goodness of fitness of the RA.

**Figure 12:**
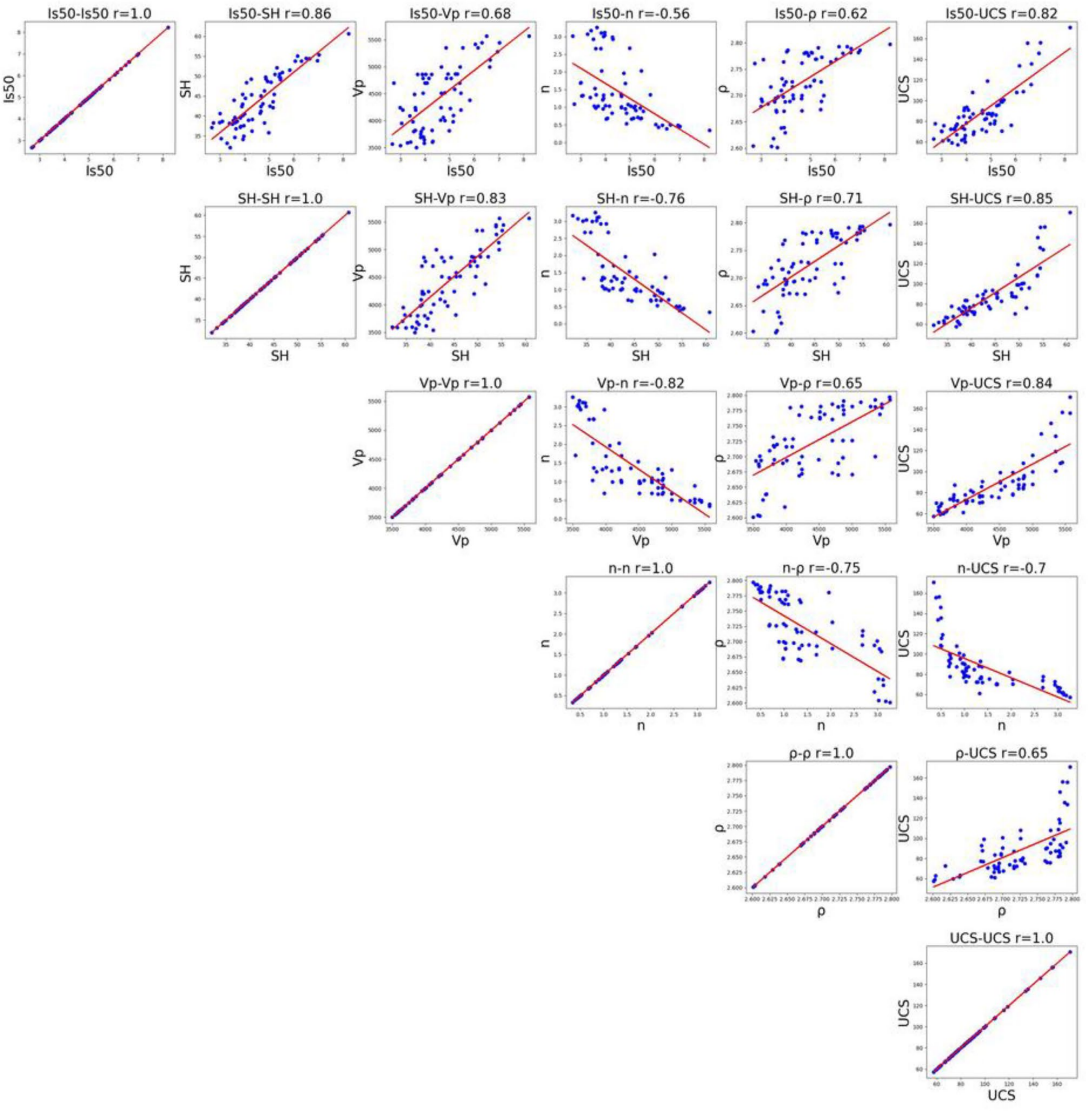
Scatterplot of weakly Sandy Dolomite in terms of UCS, *SH*, *I*_*s(50)*_,* V*_*p*_, *ρ*, and *n.*

In addition to the descriptive statistics of UCS, *SH*, *I*_*s(50)*_, *V*_*p*_, *ρ*, and *n*, the Pearson correlation coefficients between each pair of variables were calculated based on the database (Fig. 12). The five parameters are highly correlated with UCS, which implied that they have a significant correlation in predicting UCS, and the correlation matrix is shown in Table 4.

**Table 4:** Correlation matrix between UCS and PMP.

	I_s(50)_	SH	Vp	n	ρ	UCS
I_s(50)_	1.000000					
SH	0.855277	1.000000				
Vp	0.680445	0.831487	1.000000			
n	− 0.5577330	− 0.757734	− 0.818705	1.000000		
ρ	0.620034	0.713312	0.653436	− 0.747263	1.000000	
UCS	0.820452	0.852588	0.840153	− 0.699622	0.654727	1.000000

The synergetic coefficient between UCS and *SH*, *I*_*s(50)*_, *V*_*p*_, and *ρ* of weakly Sandy Dolomite is positive, which demonstrate a positive correlation between UCS and these four parameters (*SH*, *I*_*s(50)*_, *Vp*, *ρ*) and that UCS increases with the rise of four parameters. The synergetic coefficient between UCS and *n* is negative, which demonstrate that there is a negative correlation between the two, and UCS decreases with the rise of *n* (Fig. 12 and Table 4). The UCS of weakly Sandy Dolomite, five parameters exhibits good prediction performance, among which the most optimal parameters are *SH* and *I*_*s(50)*_, the effective parameters are *V*_*p*_ and *ρ* [Eq. (15) and Table 4].

### CNN training

MSE was employed as the loss function of CNN training. Both the input and output data must first be normalized so that produce a remarkable convergence effect during training. In fact, the description of MSE is actually the prediction error following normalization. Meanwhile, MAE was also used to describe the prediction error. MAE will not alter the input or output data and can accurately reflect the prediction error of UCS in standard units, and thus MAE is also utilized for final verification.

There are five input feature parameters (*SH*, *I*_*s(50)*_, *Vp*, *ρ*, *n*) in training, and the dimension is 5*1*n, *n* is the number of input data groups each time. The UCS is the output parameter and its dimension is 1*n. In order to fit the mapping relationship between the input and output parameters, two types of hidden layers were designed to ensure the degree of non-linearity in CNN. The structure of input layer + hidden layer I + hidden layer II + output layer was established, and the Bayesian optimization method was employed to search the number of neurons in the hidden layer, ensuring that the ReLU layer was used for nonlinear operation following each layer.

With different input data units, we performed normalized operations for each input so that the data values are uniformly mapping within the range of [− 1, 1]. The standardized predicted value and real value (measured value of UCS) are also used in the loss function during training to calculate MSE.

For medium Sandy Dolomite, the data of 75% (90 × 75% ≈ 67) database with a size of 90 was used for CNN training and selection, and the remaining 25% (90–67 = 23) was used for comparison and verification. For weakly Sandy Dolomite, the data of 75% (68 × 75% = 51) database with a size of 68 was used for CNN training and selection, and the remaining 25% (68–51 = 17) was used for comparison and verification.

### Comparison between RA and CNN

#### Comparison between RA and CNN of medium Sandy Dolomite

According to the CNN calculation results, the optimal CNN structure for predicting medium Sandy Dolomite data is 5–300–300–1. That is, the input layer with five parameters (*SH*, *I*_*s(50)*_, *Vp*, *ρ*, *n*) + the hidden layer I with 300 neurons + the hidden layer II with 300 neurons + the output layer with one parameter (UCS). The values of UCS in validation data set were predicted and compared with the real measured values for evaluating and comparing the performance of RA and CNN. Similarly, R^2^, MSE, RMSE, MAE, and MAPE were calculated by using Eqs. (9)–(13), as shown in Table 5.

**Table 5:** MAE, MSE, RMSE, MAPE, and R^2^ calculation results of medium Sandy Dolomite.

Model	MAE	MSE	RMSE	MAPE (%)	R^2^
RA	1.848	5.692	2.386	4.397	0.973
CNN	1.407	3.232	1.798	3.168	0.979

**Figure 13:**
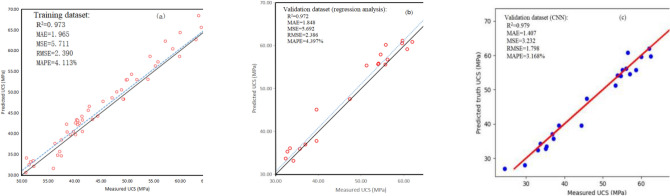
R^2^, MSE, RMSE, MAE, MAPE and mean ratio of predicted and measured UCS values. (**a**) training data set; (**b**) validation data set (RA); (**c**) validation data set (CNN).

**Figure 14:**
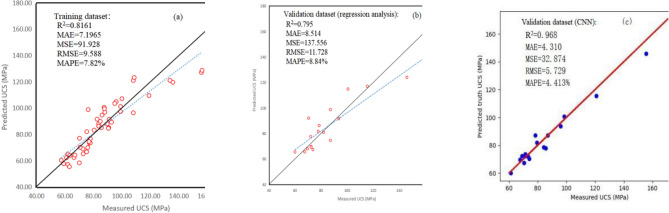
R^2^, MSE, RMSE, MAE, MAPE and mean ratio of predicted and measured UCS values. (**a**) training data set; (**b**) validation data set (RA); (**c**) validation data set (CNN).

The value of MAE, MSE, RMSE, and MAPE in CNN are all smaller than in the RA, while R^2^ of CNN is larger than that of the RA. The larger the R^2^ is, the smaller the MSE, RMSE, and MAPE are, which implied that the performance of the prediction model is better (Table 5). Therefore, the CNN is more suitable for predicting the UCS of medium Sandy Dolomite than RA due to its more precise results. The predicted and measured UCS values are displayed in Fig. 14a–c to visualize the advantages and disadvantages of RA and CNN in predicting UCS. The dot result usually fluctuates near the 1:1 line (the solid line), indicating that the training and validation data sets are suitable for the prediction of UCS (Fig. 13a,b).

The results base on validation data illustrate that the R^2^ of RA is 0.972, while the R^2^ of CNN is 0.979. Compared with the R^2^ of 0.973 based on training data, the R^2^ of CNN is 0.72% higher than that of RA. Similarly, compared with the results based on training data, the MAE, MSE, RMSE, and MAPE values of CNN are 22.45%, 43.07%, 24.60%, and 29.88% lower than those of RA, indicating that CNN has an evidently better performance than RA in terms of the UCS prediction of medium Sandy Dolomite (Fig. 13).

### Comparison between RA and CNN on weakly Sandy Dolomite

According to the CNN calculation results, the optimal CNN structure for predicting weakly Sandy Dolomite data is 5–128–224–1. That is, the input layer with five parameters (*SH*, *I*_*s(50)*_, *Vp*, *ρ*, *n*) + the hidden layer I with 128 neurons + the hidden layer II with 224 neurons + the output layer with one parameter (UCS). The values of UCS in validation data set were predicted and compared with the real measured values for evaluating and comparing the performance of RA and CNN. Similarly, R^2^, MSE, RMSE, MAE, and MAPE were calculated by using Eqs. (9)–(13), as shown in Table 6.

**Table 6:** MAE, MSE, RMSE, MAPE, and R^2^ calculation results of weakly Sandy Dolomite.

Model	MAE	MSE	RMSE	MAPE (%)	R^2^
RA	8.514	137.556	11.728	8.840	0.795
CNN	4.310	32.824	5.729	4.413	0.968

The value of MAE, MSE, RMSE, and MAPE in CNN are all smaller than in the RA, while R^2^ of CNN is larger than that of the RA. The larger the R^2^ is, the smaller the MSE, RMSE, and MAPE are, which imply that the performance of the prediction model is better (Table 6). Therefore, the CNN is more suitable for predicting the UCS of weakly Sandy Dolomite than RA due to its more precise results. The predicted and measured UCS values are displayed in Fig. 14a–c to visualize the advantages and disadvantages of RA and CNN in predicting UCS. The dot result usually fluctuates near the 1:1 line (the solid line), indicating that the training and validation data sets are suitable for the prediction of UCS (Fig. 14a,b).

The results based on validation data illustrate that the R^2^ of RA is 0.795, while the R^2^ of CNN is 0.968. Compared with the R^2^ of 0.8161 based on training data, the R^2^ of CNN is 21.2% higher than that of RA. Similarly, compared with the results based on training data, the MAE, MSE, RMSE, and MAPE values of CNN are 58.42%, 113.87%, 62.76%, and 56.61% lower than those of RA, indicating that CNN has an evidently better performance than RA in terms of the UCS prediction of weakly Sandy Dolomite (Fig. 14).

## Conclusions

In conclusion, *SH*, *I*_*s(50)*_, *Vp*, *ρ*, and *n* were utilized to predict the UCS of Sandy Dolomite, and R^2^, MSE, RMSE, MAE, and MAPE were utilized to evaluate and compare the performance of both RA and CNN in this study. The main conclusions are as follows:

1. Bayesian optimization is utilized in CNN to search the data in the hidden layer, and obtained prediction results were much higher than that of RA, indicating the reliability of the CNN-based prediction model. The prediction precision of the CNN model can be further improved as the number of indicators increases.
2. Fierce Sandy Dolomite is a rock with low density and high porosity; strongly Sandy Dolomite is a rock with medium density and porosity; medium and weakly Sandy Dolomite is a rock with high density and low porosity.
3. The sandification grade of dolomite increases with the rise of porosity and decreases with the rise of density.
4. Schmidt hammer rebound test results demonstrated that the minimum thickness of medium and weakly Sandy Dolomite is 110 mm and 75 mm, respectively.
5. As for Sandy Dolomite, there is a positive correlation between UCS and *SH*, *I*_*s(50)*_, *V*_*p*_, and *ρ*, while there is a negative correlation between UCS and *n*.

Compared with RA, CCN has the higher accuracy for predicting the UCS of Sandy Dolomite. However, the mathematic expressions between UCS and its relevant indexes are unavailable via CNN analysis. Such impact maybe come from CNN itself, in most situations, the interaction between inputs and results does not provide a deterministic pattern due to the so called “black box”. This indicates that some more straightforward and understandable methods within a full system are needed to be further researched.

## Data Availability

The datasets used and/or analysed during the current study available from the corresponding author on reasonable request.

## CYWD project

UCS: Uniaxial compressive strength
RA: Regression analysis
PMP: Petrophysical and mechanical properties
SH: Schmidt hammer rebound index
V_p_: Compressional wave velocity
n: Porosity
MSE: Mean square error
MAE: Mean absolute error
RQD: Rock quality designation
f_UCS,i_: Predicted values of UCS
m: Number of the measured values of UCS
ASTM: American society for testing and materials

## Central Yunnan water diversion project

PLT: Point load test
CNN: Convolutional neural network
ANN: Artificial neural network
I_s(__50)_: Point load index
ρ: Density
R^2^: Correlation coefficient
RMSE: Root mean square error
MAPE: Mean absolute percentage error
f_i_: Measured values of UCS
$\overline{f }$: Average of the measured UCS values
ReLU layer: Rectified linear units layer
ISRM: International society for rock mechanics
